# Real‐life treatment of cutaneous warts with cantharidin podophyllin salicylic acid solution

**DOI:** 10.1111/dth.13143

**Published:** 2019-11-20

**Authors:** Anh Ly Nguyen, Koen D. Quint, Jan Nico Bouwes Bavinck, Angelina Erceg, Wim J. A. de Kort, John E. M. Körver

**Affiliations:** ^1^ Department of Dermatology Amphia Hospital Breda The Netherlands; ^2^ Department of Dermatology Leiden University Medical Center Leiden The Netherlands; ^3^ Department of Dermatology Roosevelt Clinics Leiden The Netherlands

**Keywords:** cantharidin podophyllin salicylic acid solution, clearance rate, patient satisfaction, treatment outcome, warts

## Abstract

Patients often request treatment of their burdensome cutaneous warts. However, a safe and effective treatment for cutaneous warts is lacking. This study evaluates treatment outcome, side effects, and patient satisfaction after topical application of cantharidin 1% podophyllin 2% salicylic acid 30% (CPS1) solution in a large series of children and adults with cutaneous warts. Fifty‐two children and 83 adults with warts, treated with CPS1 solution between October 2012 and October 2014, were included. Complete clearance of warts occurred in 86.5% of children and 62.7% of adults treated with CPS1 solution (*p* < .01). Resolution of warts was partial in 3.9 and 24.1% and absent in 9.6 and 13.2% of children and adults respectively. Side effects were present in 41.2% of children and 46.3% of adults (*p* = .7). Most common side effects were blistering, pain, and burning sensation. No serious adverse events occurred. On a 10‐point scale, median patient satisfaction score was 9.0 (interquartile range 7.8–10.0) and 8.0 (interquartile range 5.1–9.7) for children and adults respectively (*p* < .01). CPS1 solution is a safe and promising treatment modality with a high clearance and high patient satisfaction rate for the management of cutaneous warts, particularly in children.

## 1 INTRODUCTION

Warts are a common viral infection of the skin caused by certain types of the human papillomavirus (HPV; Bruggink et al., 2012). As a result of natural immunity, without treatment, warts tend to resolve spontaneously within months to years (Sterling, Gibbs, Haque Hussain, Mohd Mustapa, & Handfield‐Jones, 2014). Prior research demonstrated that 52% of warts in children cleared within 1 year and two thirds of the warts resolved within 2 years, regardless of treatment of the warts (Bruggink, Eekhof, et al., 2013; Massing & Epstein, 1963). An expectant approach is often advised for the management of the cutaneous warts. However, patients often request treatment of warts due to pain, discomfort, and/or social stigma. Treatment of warts is often painful, labor intensive, time‐consuming, and expensive. A safe and effective treatment for cutaneous warts is still lacking (Sterling et al., 2014).

A wide variation of treatment modalities is available for the management of warts. The range of topical treatment modalities includes duct tape, topical salicylic acid, cryotherapy, silver nitrate, monochloroacetic acid, podophyllin, cantharidin, and 5‐fluorouracil (Bock, 1965; Bruggink et al., 2010; Bruggink et al., 2015; Cockayne et al., 2011; Duthie & McCallum, 1951; Ebrahimi, Dabiri, Jamshidnejad, & Sarkari, 2007; Epstein & Epstein, 1960; Gladsjo, Alió Sáenz, Bergman, Kricorian, & Cunningham, 2009; Kartal Durmazlar, Atacan, & Eskioglu, 2009; Kwok, Holland, & Gibbs, 2011; Rosenberg, Amonette, & Gardner, 1977; Salk, Grogan, & Chang, 2006). A meta‐analysis and pooled analysis from randomized controlled trials reported cure rates ranging from 30 to 58% following treatment of cutaneous warts with duct tape, cryotherapy, salicylic acid, topical 5‐fluorouracil, and combined therapy with salicylic acid and cryotherapy (Kwok et al., 2011). The overall evidence for most of these treatments is weak, due to difficulties in study design (Sterling et al., 2014).

In contrast to most wart treatments, the application of cantharidin solution is painless. Cantharidin is a vesicant produced by blister beetles, which has been used as a treatment of warts and molluscum contagiosum since the 1950s (Torbeck, Pan, DeMoll, & Levit, 2014). Combinations of cantharidin 1% podophyllin 2% salicylic acid 30% (CPS1) solution, cantharidin 1% podophyllotoxin 5% salicylic acid 30% (CPS2), and cantharidin 1% podophyllin 20% salicylic acid 30% (CPS3) solution are also available for the treatment of warts. Cure rates after topical application of cantharidin solution for the treatment of cutaneous warts range from 64.0 to 100% (Bock, 1965; Epstein & Epstein, 1960; Kartal Durmazlar et al., 2009; Rosenberg et al., 1977). Cure rates for the treatment of plantar warts with topical application of CPS2 and CPS3 solution are even higher, ranging from 81.0 to 100.0% (Becerro de Bengoa Vallejo, Losa Iglesias, Gómez‐Martín, Sánchez Gómez, & Sáez Crespo, 2008; Coskey, 1984; Ghonemy, 2017; Kaçar, Taşlı, Korkmaz, Ergin, & Erdoğan, 2012; López López et al., 2016; López‐López, Agrasar‐Cruz, Bautista‐Casasnovas, & Álvarez‐Castro, 2015). The study size and follow up period were often limited (Becerro de Bengoa Vallejo et al., 2008; Bock, 1965; Coskey, 1984; Epstein & Epstein, 1960; Ghonemy, 2017; Kaçar et al., 2012; Kartal Durmazlar et al., 2009; López López et al., 2016; López‐López et al., 2015; Rosenberg et al., 1977). “In some studies, the associated predicting factors enhancing the resolution of warts such as age of patients were not accounted for." (Becerro de Bengoa Vallejo et al., 2008; Bock, 1965; Epstein & Epstein, 1960; Kartal Durmazlar et al., 2009; Rosenberg et al., 1977). A previous study demonstrated that resolution of warts was more likely at a younger age (Bruggink, Eekhof, et al., 2013). Moreover, the fact that a patient‐friendly and effective treatment with painless application for warts in children is still lacking, calls for a study investigating treatment with topical application of CPS1 solution for cutaneous warts in children versus adults.

This study aimed to evaluate outcome, patient satisfaction, and side effects of cutaneous warts treated with topical CPS1 solution in a large series of children and adults.

## 2 MATERIALS AND METHODS

### 2.1 Study cohort

This retrospective study was conducted at a secondary non‐university teaching hospital in Breda, The Netherlands. The ethics committee of Medical Research Ethics Committees United exempted this study for review, as it was a survey‐based retrospective study (W16.037). Therefore, this research was not subject to the Medical Research Involving Human Subjects Act. Patients who received treatment with topical application of CPS1 solution for cutaneous warts between October 2012 and October 2014 were eligible for inclusion. Exclusion criteria were plane or anogenital warts, seborrheic keratoses, immune compromised patients, pregnant, or lactating women.

### 2.2 Treatment and definitions

Treatments were performed by trained health care professionals at our outpatient clinic, under the supervision of a physician. Self‐application of CPS1 solution was not allowed due to its toxicity and potential misuse, as it can be fatal if ingested (Torbeck et al., 2014). Excessive callus was removed by using a curette or scalpel. The treatment regimen consisted of topical application of cantharidin 0.7% solution followed by subsequent applications with CPS1 solution. For the first treatment, cantharidin 0.7% solution was applied in order to carefully assess the extent of blister formation and other side effects. The solution was applied to the wart with a 1‐mm surrounding rim of normal skin. Treated warts were covered with occlusive tape. Patients were advised to rinse the solution off the treated warts 4–8 hr after application (in accordance with the product instruction), once a tingling/burning sensation, or blister appeared. Application of the solutions was performed at intervals of 3–4 weeks until complete clearance of all treated warts had occurred. Complete clearance was defined as resolution of all treated warts, noted by physician and/or patient. Clearance was considered partial when a section of treated warts had disappeared or if treated warts became smaller compared to baseline, based on patient's experience. Warts that initially cleared with treatment, but reoccurred at the same location within 3 years of treatment with CPS1 solution were noted as recurrences.

### 2.3 Data collection

Data were collected from a combination of electronic patient records and a survey. The physician judged clearance of the warts during regular check‐ups. In case of discrepancies between the electronic patient records and the survey, the information from the electronic patient records was used to minimize recall bias. Patients were approached by post or telephone to complete the survey. The survey included questions regarding the time span of the warts before treatment with CPS1 solution, reasons for desiring treatment of warts, any wart treatment before and after treatment with CPS1 solution, side effects after application of CPS1 solution, clearance of all treated warts, recurrences, and patient satisfaction regarding treatment with CPS1 solution. The patient satisfaction score was measured on a 10‐point scale; the higher the score, the higher the level patient satisfaction. A serious adverse event was defined as a life‐threatening adverse event, resulting in death, requiring inpatient hospitalization, prolongation of hospitalization or an event resulting in persistent or significant disability or incapacity. Upon request, an English translation of the survey is available from the corresponding author. In reporting this study, we adhered to the Strengthening the Reporting of Observational Studies in Epidemiology statement.

### 2.4 Statistical analysis

Continuous variables with a non‐normal distribution were summarized as median with a corresponding interquartile range (IQR). Categorical variables were summarized as frequencies and percentages. Statistical analysis of continuous variables was performed using the Pearson chi‐square test and for statistical analysis of two independent groups on a continuous variable, the Mann–Whitney *U* test was performed. In case of categorical variables with three or more categories, the Kruskal–Wallis test was applied. Outcomes were considered statistically significant if *p* **<** .05. In case of missing data, pairwise deletion was performed. The data analyses were performed with SPSS Statistics Pack version 25.0 (IBM, Armonk, NY).

## 3 RESULTS

### 3.1 Patient characteristics

A total of 147 patients were treated with topical application of CPS1 solution for cutaneous warts between October 2012 and October 2014. Five patients declined to participate in the survey and six patients were lost to follow‐up. One patient was excluded as the survey was completed anonymously. A total of 52 children (age < 18 years) with the help of their parents and 83 adults (age ≥ 18 years) completed the survey and were included in the analyses.

Overall, a majority of the patients were adults (61.5%), female (53.3%), and had more than one wart (69.2%). Adults more often had plantar warts in comparison to children (61.4 vs. 44.2%, *p* = .05). The majority of children had common warts. Warts in children were twice as likely to have been present for <12 months at first presentation compared to adults (46.0 vs. 23.4%, *p* < .01). Children more frequently had multiple reasons for desiring treatment in comparison to adults (74.5 and 54.2%, respectively, *p* = .05). Children experienced social and cosmetic inconveniences of the warts twice as often as adults. Only 3.8% of children and 4.8% of adults had not received previous treatment for their warts. There were no significant differences between children and adults in terms of number of treatments prior, during, or after therapy with CPS1 solution (Supplementary Table). The median follow‐up period was 28.3 months with IQR of 18.8–32.5 months.

### 3.2 Treatment outcome, patient satisfaction and side effects

Clearance of warts was complete in 86.5% *(95% confidence interval [CI]*: *74.7%*; *93.3%)* of children and 62.7% *(95% CI*: *51.9%*; *72.3%)* of adults treated with CPS1 solution. Resolution of warts was partial in 3.9% *(95% CI*: *1.1%*; *13.0%)* and 24.1% *(95% CI*: *16.2%*; *34.3%)* and ineffective in 9.6% *(95% CI*: *4.2%*; *20.6%)* and 13.2% *(95% CI*: *7.6%*; *22.2%)* of children and adults respectively (*p* < .01). Children required significantly fewer treatments with CPS1 solution compared to adults: 65.4% of children received 1–3 treatments with CPS1 solution as opposed to 37.3% of adults (*p* < .01). A majority of the adults (62.7%) required ≥4 treatments with CPS1 solution for their warts. Children were completely cleared from their warts at a median of 3.0 months (IQR 1.6–6.0) after onset of treatment with CPS1 solution, while for adults complete clearance occurred at a median of 5.0 months (IQR 3.0–6.0; *p* = .02). Of the warts that initially cleared with CPS1 solution, recurrence of the warts occurred in 10.6% of children and 19.4% of adults (*p* = .3) (Table 1).

**Table 1:** Treatment outcome and patient satisfaction after topical application of CPS1 solution for cutaneous warts

	Children (age <18)	Adults (age ≥18)	Total	*p* Value
Clearance of warts#	*n* = 52	*n* = 83	*n* = 135	<.01*
Complete	45 (86.5)	52 (62.7)	97 (71.9)	
Partial	2 (3.9)	20 (24.1)	22 (16.3)	
None	5 (9.6)	11 (13.2)	16 (11.8)	
Number of treatments with CPS1 solution~	*n* = 52	*n* = 83	*n* = 135	<.01*
1–3	34 (65.4)	31 (37.3)	65 (48.1)	
≥ 4	18 (34.6)	52 (62.7)	70 (51.9)	
Number of treatments with CPS1 solution~, median (IQR)	3.0 (2.0–4.0)	4.0 (3.0–5.0)	4.0 (2.0–5.0)	<.01*
Duration before clearance of warts after onset treatment with CPS1 in months#, median (IQR)	3.0 (1.6–6.0)	5.0 (3.0–6.0)	4.0 (2.5–6.0)	.02*
Recurrence of warts#	*n* = 47	*n* = 72	*n* = 119	.3
Yes (complete/partial)	5 (10.6)	14 (19.4)	19 (16.0)	
No	42 (89.4)	58 (80.6)	100 (84.0)	
Patient global assessment of treatment effect∞	*n* = 51	*n* = 83	*n* = 134	.01*
Good, very good or excellent	45 (88.2)	57 (68.7)	102 (76.1)	
Bad or reasonable	6 (11.8)	26 (31.3)	32 (23.9)	
Patient satisfaction score∞, median (IQR)	9.0 (7.8–10.0)	8.0 (5.1–9.7)	8.5 (6.0–9.95)	<.01*

*Note*: Data are *n* (%) unless stated otherwise. Abbreviations: CPS1, cantharidin 1% podophyllin 2% salicylic acid 30%; IQR, interquartile range; *n*, total number of patients; *, *p* value **<**.05; ~, data obtained from patients' electronic record; #, data obtained from patients' electronic record and survey; ∞, data obtained from survey.

A majority of children and adults rated the effect of treatment with CPS1 solution as good, very good or excellent (88.2 vs. 68.7% respectively, *p* = .01). The median patient satisfaction score was higher for children than adults, namely 9.0 (IQR 7.8–10.0) versus 8.0 (IQR 5.1–9.7), respectively, (*p* < .01; Table 1).

Side effects of treatment with CPS1 solution were present in 41.2% of the children and 46.3% of adults (*p* = .7). The most common side effects were blistering, pain, and burning sensation. No serious adverse events occurred (Table 2).

**Table 2:** Side effects due to topical application of CPS1 solution for cutaneous warts

	Children (age < 18)	Adults (age ≥ 18)	Total	*p* value
Number of side effects#	*n* = 51	*n* = 82	*n* = 133	.7
None	30 (58.8)	44 (53.7)	74 (55.6)	
1	11 (21.6)	17 (20.7)	28 (21.1)	
2	4 (7.8)	12 (14.6)	16 (12.0)	
≥ 3	6 (11.8)	9 (11.0)	15 (11.3)	
Type of side effects#^	*n* = 50	*n* = 81	*n* = 131	NC
Blistering	44 (88.0)	77 (95.1)	121 (92.4)	
	*n* = 51	*n* = 82	*n* = 133	NC
Pain	12 (23.5)	32 (39.0)	44 (33.1)	
Burning sensation	6 (11.8)	19 (23.2)	25 (18.8)	
Irritation of skin	4 (7.8)	7 (8.5)	11 (8.3)	
Erythema	6 (11.8)	4 (4.9)	10 (7.5)	
Pigmentation of skin	2 (3.9)	5 (6.1)	7 (5.3)	
Itching	3 (5.9)	1 (1.2)	4 (3.0)	
Scarring	2 (3.9)	0 (0.0)	2 (1.5)	
Infection of skin	0 (0.0)	0 (0.0)	0 (0.0)	
Other	4 (7.8)	7 (8.5)	11 (8.3)	

*Note*: Data are *n* (%). Abbreviations: CPS1, cantharidin 1% podophyllin 2% salicylic acid 30%; *n*, total number of patients; NC, not calculable due to multiple answer options; #, data obtained from patients' electronic record and survey; ^, Multiple answers are possible.

Subgroup analysis based on location of the warts (plantar warts, common warts, combined plantar, and common warts) showed no significant difference in terms of treatment outcomes.

## 4 DISCUSSION

This study reports the largest series of patients with warts treated with CPS1 solution to date. This study demonstrated a high complete clearance rate (86.5 and 62.7%, *p* < .01) with only mild side effects and a high degree of patient satisfaction (88.2 and 68.7%, *p* < .01) of cutaneous warts treated with CPS1 solution in children and adults, respectively.

Previous studies reported somewhat higher clearance rates ranging from 81.0 to 100.0% after treatment of plantar warts with topical application of CPS2 and CPS3 solution. However, there are some clear differences in treatment design and secondary outcomes among these studies. For one, the duration of occlusion in these studies was significantly longer and varied from 24 to 48 hr with weekly to monthly applications with CPS2 or CPS3 solution (Table 3). Furthermore, podophyllotoxin 5% and podophyllin 20% were used as a component of the CPS2 and CPS3 solution respectively as opposed to podophyllin 2% in the CPS1 solution we applied in this study (Becerro de Bengoa Vallejo et al., 2008; Coskey, 1984; Ghonemy, 2017; Kaçar et al., 2012; López López et al., 2016; López‐López et al., 2015). It is likely that longer duration of occlusion, shorter treatment intervals and higher concentration of the components of the CPS2 and CPS3 solution somewhat enhanced resolution of the warts. However, this relatively modest increase in clearance rate did come at a cost. The studies using CPS2 solution reported significantly more severe side effects and higher toxicity than our study, using CPS1 solution (Becerro de Bengoa Vallejo et al., 2008; Coskey, 1984; Kaçar et al., 2012; López López et al., 2016; López‐López et al., 2015). One study reported cellulitis in 3.3% of children treated with topical application of CPS2 solution followed by an occlusion period of 24 hr (Coskey, 1984). Another study more frequently reported pain following treatment of the warts with the CPS2 solution as opposed to the CPS1 solution in our study (85.7 vs. 33.1%; Kaçar et al., 2012). Treatment of plantar warts with CPS3 solution caused complications of pain, bulla, and hemorrhagic bulla in 26.7, 60, and 13.3% of the patients (Ghonemy, 2017). We adhered to the product instruction of CPS1 solution, which advised an occlusion period of 4–8 hr. Our study reported only mild side effects, no secondary infections, or serious adverse events. Furthermore, these mild side effects in our study also coincided with a high patient satisfaction rate. Therefore, we consider topical application of CPS1 solution followed by an occlusion period of 4–8 hr as an effective, safe, and well‐tolerated treatment for cutaneous warts, especially in children.

**Table 3:** Overview of studies regarding treatment of cutaneous warts with topical application of CPS1, CPS2, or CPS3 solution

	Study design	Patient group	*N* patients (*N* warts)	Treatment	Duration of occlusion	Interval	*N* patients with cleared warts/*N* patients reached at follow up	Efficacy rate (%)	Recurrences *n* patients (%)	Follow‐up period
Coskey (1984)	P	Children	121 (NR)	CPS2	24 hr	Once weekly	81/100	81.0	NR	6–12 months
Becerro de Bengoa Vallejo et al. (2008)	R	Children and adults	144 (NR)	CPS2	24–48 hr	Every 2–3 weeks	138/144	95.8	0/144 (0)	6 months
Kaçar et al. (2012)	RCT	Adults	14 (75)	CPS2	24 hr	Every 2 weeks	14/14	100.0	0/14 (0)	NR
López López et al. (2016)	P	Children and adults	75 (126)	CPS2	24–48 hr	After 15–20 days	75/75	100.0	NR	NR
Ghonemy (2017)	P	Adults	15 (78)	CPS3	24 hr	Every 2 weeks	14/15	93.3	NR	6 months
Current study	R	Children versus adults	135 (NR)	CPS1	4–8 hr	Every 3–4 weeks	97/135	71.9	19/119 (16.0)	8–40 months

*Note*: Data are *n* (%) unless stated otherwise. Abbreviations: CPS1, cantharidin 1% podophyllin 2% salicylic acid 30%; CPS2, cantharidin 1% podophyllotoxin 5% salicylic acid 30%; CPS3, cantharidin 1% podophyllin 20% salicylic acid 30%; *N*, number; NR, not reported; P, prospective cohort study; R, retrospective cohort study; RCT, randomized controlled trial.

This was the first study evaluating treatment outcomes after topical application of CPS1 solution for the management of cutaneous warts in children versus adults. As this study shows, children more often than adults seek treatment for warts due to social and cosmetic inconveniences. Children are more difficult to treat as painful treatments are poorly tolerated and should preferably be avoided (Sterling et al., 2014). In daily practice, this can be challenging for both doctor and patient. Therefore, a distinction between children and adults is crucial in evaluating and choosing an adequate, satisfactory, and effective topical treatment for cutaneous warts (Bruggink, Eekhof, et al., 2013; Sterling et al., 2014). A review of the management of cutaneous warts in children proposed salicylic acid 25–35% as the preferred treatment for children with large, multiple, or periungual warts due to easy daily application at home and mild side effects. Cryotherapy was viewed as another simple and easy treatment, however often poorly tolerated by children due to pain, scarring and post‐inflammatory hyper‐ or hypopigmentation. They also considered cantharidin to be a valuable option for the treatment of cutaneous warts in children (Gerlero & Hernández‐Martín, 2017). The current study shows that CPS1 solution is a safe, rather quick, well tolerated and highly appreciated treatment for warts in children. However, application of the CPS1 solution by trained health care professionals is mandated (Torbeck et al., 2014). Still, costs for treatment of warts could be greatly reduced, as only limited treatment sessions (median number of 3) are needed to achieve complete resolution of warts in children compared to cryotherapy. In addition, the recurrences reported for CPS1 solution (10.6 and 19.4% for children and adults, respectively) are roughly lower or comparable to other treatments of cutaneous warts with duct tape (75%), cryotherapy (13.3–25%), 5% 5‐fluorouracil (13–15%), and pulsed dye laser (0–36%; Dhar, Rashid, Islam, & Bhuiyan, 2009; Gladsjo et al., 2009; Salk et al., 2006; Veitch, Kravvas, & Al‐Niaimi, 2017; Wenner et al., 2007; Youn, Kwon, Park, Kim, & Kim, 2011). Two studies reported no recurrences after treatment of cutaneous warts with CPS2 solution (Table 3) (Becerro de Bengoa Vallejo et al., 2008; Kaçar et al., 2012). Recurrences of cutaneous warts after topical treatment with salicylic acid are unknown. As the protocol for treatment with CPS1 solution is rather simple, one could wonder about the place of this treatment in the therapeutic arsenal of cutaneous warts. CPS1 solution could be a promising second line treatment for cutaneous warts in hospitals (and primary care) following ineffective first line treatment with salicylic acid, particularly in children.

The results in this study are real‐life, generalizable, and representative for a population of patients in a secondary hospital, as this study included a large non‐selected sample of patients with burdensome recalcitrant warts in a single center secondary hospital.

Limitations of this study include its retrospective nature, lack of a placebo group, and potential recall bias due to long follow‐up period. In this study, we tried to limit the recall bias by relying on electronic patients' records in case of discrepancies. Selection bias might also have been present, as it was probable that patients with painful or recalcitrant warts were more frequently referred to our secondary hospital than treated in primary care. These results might underestimate the true clearance rate, if the CPS1 solution were to be applied in patients with warts in a primary care setting. Additionally, previous research established that the HPV type in warts can also predict the response to certain treatments (Bruggink, Gussekloo, et al., 2013). In this retrospective study, the HPV types in warts were not tested and therefore the role of HPV type on clearance of warts in this study population remains unknown.

## 5 CONCLUSION

This study established topical application of CPS1 solution as a safe, effective, and promising treatment modality for the management of cutaneous warts, especially in children. A large prospective (placebo controlled) randomized trial is indicated to further assess this promising safe and effective treatment for cutaneous warts.

## DISCLOSURE OF INTEREST

The authors report no conflict of interest.

## Supporting information


**SUPPLEMENTARY TABLE**: Patient characteristics
