# Supplementary material for: Real‐life treatment of cutaneous warts with cantharidin podophyllin salicylic acid solution
Source: Dermatol Ther. 2019 Nov 20;32(6):e13143. doi: 10.1111/dth.13143 (PMC6916542; doi:10.1111/dth.13143)
Supplement: Supplementary file 1 — SUPPLEMENTARY TABLE: Patient characteristics [file DTH-32-na-s001.pdf]

SUPPLEMENTARY TABLE

Patient characteristics

	Children (age < 18)	Adults (age ≥ 18)	Total	*P* value
**Sex~**	n = 52	n = 83	n = 135	0.4
Female	25 (48.1)	47 (56.6)	72 (53.3)	
**Number of warts~**	n = 50	n = 83	n = 133	0.3
1	14 (28.0)	27 (32.5)	41 (30.8)	
2-5	19 (38.0)	38 (45.8)	57 (42.9)	
> 5	17 (34.0)	18 (21.7)	35 (26.3)	
**Number of anatomic sites involved~**	n = 52	n = 83	n = 135	< 0.01*
1	31 (59.6)	70 (84.3)	101 (74.8)	
≥ 2	21 (40.4)	13 (15.7)	34 (25.2)	
**Location of warts~^**				NC
Common warts	47 (90.4)	38 (45.8)	85 (63.0)	
Plantar warts	23 (44.2)	51 (61.4)	74 (54.8)	
Peri-ungual warts	9 (17.3)	10 (12.0)	19 (14.1)	
Other	3 (5.8)	0 (0.0)	3 (2.2)	
**Duration of warts#**	n = 50	n = 81	n = 131	< 0.01*
≤ 6 months	8 (16.0)	13 (16.0)	21 (16.0)	
6-12 months	15 (30.0)	6 (7.4)	21 (16.0)	
12-24 months	14 (28.0)	21 (25.9)	35 (26.7)	
> 24 months	13 (26.0)	41 (50.6)	54 (41.2)	
**Number of reasons for desiring treatment of warts#**	n = 51	n = 83	n = 134	0.05
1	13 (25.5)	38 (45.8)	51 (38.1)	
2	29 (56.9)	32 (38.5)	61 (45.5)	
≥ 3	9 (17.6)	13 (15.7)	22 (16.4)	
**Reason for desiring treatment of warts#^**				NC
Pain	19 (37.3)	48 (57.8)	67 (50.0)	
Discomfort	31 (60.8)	59 (71.1)	90 (67.2)	
Social inconvenience	21 (41.2)	14 (16.9)	35 (26.1)	
Cosmetic inconvenience	25 (49.0)	21 (25.3)	46 (34.3)	
Other	3 (5.9)	3 (3.6)	6 (4.5)	
**Number of previous treatments#**	n = 52	n = 83	n = 135	0.6
0-1	19 (36.5)	26 (31.3)	45 (33.3)	
≥ 2	33 (63.5)	57 (68.7)	90 (66.7)	
**Previous treatments of the warts#^**				NC
Salicylic acid	13 (25.0)	24 (28.9)	37 (27.4)	
Ditranol salicylic acid	24 (46.2)	35 (42.2)	59 (43.7)	
Cryotherapy	43 (82.7)	72 (86.7)	115 (85.2)	
Other	13 (25.0)	35 (42.2)	48 (35.6)	
**Concurrent treatments of the warts~**	n = 52	n = 83	n = 135	0.3
No	48 (92.3)	71 (85.5)	119 (88.1)	
Yes (1 or 2 treatments)	4 (7.7)	12 (14.5)	16 (11.9)	
**Concurrent treatments of the warts~^**				NC
Salicylic acid	1 (1.9)	2 (2.4)	3 (2.2)	
Ditranol salicylic acid	1 (1.9)	8 (9.6)	9 (6.7)	
Cryotherapy	2 (3.8)	3 (3.6)	5 (3.7)	
Other	0 (0.0)	1 (1.2)	1 (0.7)	
**Treatments after CPS1 solution#**	n = 51	n= 83	n = 134	0.2
No	41 (80.4)	58 (69.9)	99 (73.9)	
Yes (1-3 treatments)	10 (19.6)	25 (30.1)	35 (26.1)	
**Other treatment of the warts after CPS1 solution#^**				NC
Salicylic acid	1 (2.0)	1 (1.2)	2 (1.5)	
Ditranol salicylic acid	3 (5.9)	13 (15.7)	16 (11.9)	
Cryotherapy	4 (7.8)	12 (14.4)	16 (11.9)	
Other	2 (3.9)	9 (10.8)	11 (8.2)	

Data are n (%). Abbreviations: CPS1, Cantharidin 1% podophyllin 2% salicylic acid 30%; n, Total number of patients; NC, Not calculable due to multiple answer options; ~, data obtained from patients' electronic record; #, data obtained from patients' electronic record and survey; ^, Multiple answers are possible; *, P value < 0.05
